# Electroporation of Mouse Follicles, Oocytes and Embryos without Manipulating Zona Pellucida

**DOI:** 10.3390/jdb9020013

**Published:** 2021-04-01

**Authors:** Bilal Ahmad Hakim, Vaishali Tyagi, Saurabh Kumar Agnihotri, Amar Nath, Ankit Kumar Agrawal, Ankita Jain, Deependra Singh, Rituraj Konwar, Monika Sachdev

**Affiliations:** 1 Division of Endocrinology, CSIR-Central Drug Research Institute (CDRI), Sector 10, Jankipuram Extension, Lucknow 226031, India; bilal.cdri@gmail.com (B.A.H.); vaishalityagimzn91@gmail.com (V.T.); saurabh0086@gmail.com (S.K.A.); amar.katiyar@gmail.com (A.N.); agarwalankit265@gmail.com (A.K.A.); ankita.cdri@gmail.com (A.J.); deependrasingh300@gmail.com (D.S.); rituraj.konwar@gmail.com (R.K.); 2 Academy of Scientific and Innovative Research (AcSIR), New Delhi 110001, India

**Keywords:** electroporation, transfection, follicle, oocyte, embryo

## Abstract

Electroporation is an effective technique of transfection, but its efficiency depends on the optimization of various parameters. In this study, a simplified and efficient method of gene manipulation was standardized through electroporation to introduce a recombinant green fluorescent protein (GFP) construct as well as RNA-inhibitors in intact mouse follicles, oocytes and early embryos, where various electroporation parameters like voltage, pulse number and pulse duration were standardized. Electroporated preantral follicles were cultured further in vitro to obtain mature oocytes and their viability was confirmed through the localization of a known oocyte maturation marker, ovastacin, which appeared to be similar to the in vivo-derived mature oocytes and thus proved the viability of the in vitro matured oocytes after electroporation. Standardized electroporation parameters, i.e., three pulses of 30 V for 1 millisecond at an interval of 10 s, were applied to manipulate the expression of mmu-miR-26a in preantral follicles through the electroporation of miR inhibitors and mimics. The TUNEL apoptosis assay confirmed the normal development of the electroporated embryos when compared to the normal embryos. Conclusively, for the first time, this study demonstrated the delivery of exogenous oligonucleotides into intact mouse follicles, oocytes and embryos without hampering their zona pellucida (ZP) and further development.

## 1. Introduction

Electroporation is an effective non-viral technique that breaches the cell membrane [1] and continues to be accepted as a delivery system at the cellular level for many molecules like DNA, dsRNA, siRNA, mRNA, proteins, peptides, antibodies and drugs [2,3,4,5,6,7,8,9,10,11]. Since its inception, various techniques have been attempted to improve the transfection efficiencies in various cell types by optimizing the transfection parameters, including voltage, pulse duration, number of pulses and pulse interval. In the present study, the fluorescence expression property of green fluorescent protein (GFP) was explored for evaluation and assessment of transfection efficiency. GFP acts as an energy-transfer acceptor and transduces the blue chemiluminescence of the protein aequorin into green fluorescent light by energy transfer. For the first time, we optimized electroporation parameters and demonstrated delivery of DNA/RNA across the zona pellucida (ZP), along with increased molecular uptake, while maintaining cell viability.

Reproduction is an essential progressive process for the survival and conservation of all living organisms. In females, it requires a developmentally competent oocyte that develops within the follicle inside the ovary. Ovarian follicle development commences with the formation of primordial follicles, which further advances to subsequent stages by changing the shape and expanding the granulosa cell network around the oocyte. During this development, a thick layer of glycoprotein, namely the zona pellicuda (ZP), takes up space between the oocyte and granulosa cells. The ZP surrounds oocytes and embryos until the early blastocyst stage, after which its hatching occurs to allow the embryo to implant [12]. Thus, the ZP attributes oogenesis, fertilization and preimplantation development [13,14]. Apart from this, the ZP acts as a natural barrier against untoward chemical and biological agents such as viruses [6]. However, this barrier is capable of reducing the efficacy of available somatic cell transfection methods that aid in deciphering the function of genes and proteins and hence precluding scientific interventions.

In order to gain insights into biological processes and molecular functions during folliculogenesis, oocyte maturation as well as early embryonic development, it is important to alter gene expression either at the transcriptional or translational level in target cells. For this, a microinjection technique has been utilized to deliver foreign genes into oocytes, zygotes and embryos by traversing the ZP layer. Even though microinjection is useful, it is difficult, tedious, low in throughput, and requires expensive equipment. Other gene delivery methods like liposomal, cationic polymer-based, virus-mediated and electroporation have also been attempted but with limited success [15]. Some studies have successfully transfected exogenous vector DNAs via electroporation in oocytes and zygotes but this required removal or weakening of the ZP with harsh acid Tyrode’s solution [16,17]. In view of the points mentioned above, this study was carried out to find out whether in vitro electroporation alone can be sufficient to deliver foreign genes to intact follicles, oocytes, and embryos without the ZP weakening treatments.

## 2. Materials and Methods

### 2.1. Follicle Collection

Follicles were collected from ovaries of 8-week female Swiss mice aseptically followed by digestion in collagenase (1 mg/mL) and DNase-I (0.2 mg/mL) in Leibovitz-15 (L-15) medium at 37 °C for 20 min. Repeat pipetting was utilized every 8–10 min to facilitate mechanical dissociation of each follicle. The criteria adopted for selection of preantral follicles was determined by their morphology, i.e., having centralized round oocytes surrounded by 2–3 layers of granulosa cells. Overall, it should be a structurally undamaged round follicular structure with a diameter of 100–120 μm [18]. Further, these designated follicles were mixed and distributed into randomized groups for the in vitro transfection experiments.

### 2.2. In Vitro Culture of Mice Preantral Follicles and Their Ovulation Induction

Following transfection via electroporation, follicles were grown in α-MEM media supplemented with 5% FBS, 8 μg/mL insulin,10 mIU/mL LH and 100 mIU/mL FSH, 100 μg/mL penicillin and 50 μg/mL streptomycin as drop culture (10 follicles/30 µL medium drop) in 35-mm culture dishes under mineral oil. After 48 h of culture, these follicles were washed three times in phosphate buffer saline (PBS) and then fixed for the assessment of transfection efficiency experiment. For the in vitro oocyte maturation experiment, follicles were maintained at 37 °C with 5% CO_2_ for 12 days. Every second day, half of the medium was replenished with the new medium. Following 12 days of culture, the follicles were further allowed to mature in the medium above containing 1.5 IU/mL human chorionic gonadotrophin (hCG) and 5 ng/mL epidermal growth factor (EGF) for 16 h [19]. The cumulus-oocyte complexes (COC) were denuded using hyaluronidase enzyme and the oocytes were monitored under an inverted microscope (Zeiss, Goettingen, Germany). These oocytes were further utilized for immuno-localization of maturation markers.

### 2.3. Oocyte and Embryo Collection

Mature (MII) oocytes and embryos were collected from 8–9-week-old super-ovulated swiss mice. The super-ovulation was induced by an injection of 5 IU pregnant mare serum gonadotropin (PMSG, Intervet, Milton Keynes, UK) and, after 48 h of PMSG injection, 5 IU human chorionic gonadotrophin (hCG, Intervet, UK) injection. COCs were collected from oviducts in mineral oil at 12–16 h after hCG injection and surrounding cumulus cells were digested by hyaluronidase. Cumulus cell free oocytes were collected and washed thrice with PBS with 1% bovine serum albumin (BSA) and used in the further experiment.

For embryo collection, super-ovulated female mice were caged with fertile male post hCG injection. Mating was secured by the presence or absence of vaginal plug and day zero of pregnancy was noted. Then, embryos were collected by flushing them out from the oviducts and uterine horn periodically at day two, three and four of gestation. The collected embryos were selected randomly for electroporation and apoptosis experiments. Mouse embryos collected on day 2 and 3 post coitum were classified as early (2–4 cell stage) and late (morula stage) embryos, respectively. The embryos collected on day 4 (blastocyst stage) were used as the control group in the TUNEL assay experiment.

### 2.4. Optimization of Electroporation Parameters in Follicle, Oocyte and Various Embryonic Stages

To measure the impact of voltage and pulse number on the transfection of green fluorescent protein-expressing construct (pEGFP-C1 Vector), 0.5 μg of purified plasmid was mixed into 20 μL of HEPES- buffered saline (150 mM NaCl and 20 mM Hepes) containing 30–80 cells from each cell type. The mixture was electroporated in the 1mm gap cuvettes with a single 1 millisecond (ms) pulse of 30, 60, 90 and 120 Volts (V) by Gene Pulser Mxcell System (Bio-rad Laboratories, Hercules, CA, USA). As per the results of a single pulse, 30 V was found to be optimal voltage with minimal effect on cellular morphology as well as on its development. Consequently, multiple pulses (2, 3, 4 and 5) of 1 ms at a gap of 10 s were evaluated in order to increase the transfection efficiency regardless of an uncompromised cellular developmental potential. Then, these follicles, oocytes and embryos were allowed to grow for 48 h in their respective culture media.

### 2.5. Assessment of Transfection Efficiency

Electroporated follicles, oocytes and embryos were cultured for 48 h, then fixed using 3.7% paraformaldehyde for 20 min at room temperature. Imaging of GFP expression was done using a confocal fluorescence microscope (Olympus, Southborough, MA, USA). Furthermore, GFP intensities of these transfects were measured by using ImageJ software (NIH, Bethesda, MD, USA). The experiments were done in triplicate and a minimum of four samples from each transfected group of the follicles, oocytes and embryos were observed for fluorescent intensities after transfection with their fluorescence intensities plotted as average intensity fluorescence intensities ± SEM.

### 2.6. Immunofluorescence of Oocyte Maturation Marker

Ovastacin (also known as sperm acrosomal SLIP1 binding protein, SAS1B), an oocyte maturation marker, was localized on in vitro matured oocytes grown from preantral follicles following electroporation [19,20]. The oocytes were collected at day 11/13 from culture, as well as after superovulation. Further, these oocytes were fixed with 3.7% paraformaldehyde in phosphate-buffered saline (PBS) for 15 min and permeabilized by 0.1% Triton-X in PBS for 30 min at 37 °C. Subsequently, the permeabilized oocytes were blocked in 3% bovine serum albumin (BSA) in PBS for 30 min at 37 °C and then incubated with the anti-SAS1B primary antibody (1:200; received as a gift from UVA, Charlottesville) in PBS with 1.5% BSA overnight at 4 °C. Following incubation, oocytes were washed thrice in washing buffer (PBS containing 1% BSA) and incubated with anti-guinea pig IgG Cy3 labeled secondary antibody (1:500; Jackson Immuno Research, West Grove, PA, USA) in the dark for 1 h at 37 °C. The oocytes were further co-stained with DNA binding dye Hoechst 33342 (Invitrogen, Carlsbad, CA, USA) for 15 min at 37 °C. Then the oocytes were washed again three times in washing buffer before mounting them on a glass slide. The samples were observed under a confocal laser-scanning microscope (Zeiss, Goettingen, Germany).

### 2.7. Assessment of Embryo Damage after Electroporation

A TUNEL (terminal deoxyuridine triphosphate nick end labelling) assay was performed to detect the embryo damage after electroporation by the Click-iTTM Plus TUNEL Assay for in situ Apoptosis Detection Kit (Invitrogen, Carlsbad, CA, USA) according to the manufacturer’s protocol. Briefly, fixed embryos were permeabilized by 0.1% Triton-X in PBS for 30 min at 37 °C. Before the terminal deoxynucleotidyl transferase (TdT) reaction initiation, positive control embryos were incubated with 1 unit of DNase I to induce DNA strand breaks for 30 min at 37 °C and washed with deionized water. Now these embryos were equilibrated with TdT reaction buffer for 10 min at 37 °C and further incubated in TdT reaction mixture for 1 h at 37 °C. Immediately after the TdT reaction, embryos were washed and incubated in the Click-iT™ Plus TUNEL reaction cocktail for 30 min in the dark at 37 °C. Hoechst 33342 solution was used to label the embryo nuclei for 15 min at 37 °C. The standard washing was done three times before each step with PBS containing 3% BSA, otherwise stated. Then the whole mount embryos were analyzed with confocal microscopy.

### 2.8. miRNA Transfection and Real-Time RT-PCR

The miR-26a mimic, an inhibitor as well as non-targeted control (Qiagen, Valencia, CA, USA), was transfected in approximately 90–100 preantral follicles in each group using best-optimized electroporation conditions, i.e., 3 pulses at 30 V, and the experiment was repeated three times. For miRNA expression, total RNA was isolated using Trizol reagent (Invitrogen, Carlsbad, CA, USA) from the pooled follicles after 48 h of culture. RNA samples were subjected to reverse transcription using the miScript II RT Kit (Qiagen;#218161), followed by quantitative real-time PCR with miScript SYBR Green PCR Kit (Qiagen; #218073) with miScript Primer assays (Qiagen; #218200) for miR-26a and RNU6-6P on the LightCycler 480 real-time PCR (Roche Diagnostics, Mannheim, Germany) according to the manufacturer’s instruction. The reference miRNA RNU6-6P values were used for the normalization of miR-26a expression.

### 2.9. Statistical Analysis

Survival percentage of preantral follicles, oocytes, early and late embryos was calculated using pooled data from at least five independent experiments. These experimental results were analyzed through the χ2 test to compare the survival rate. One-way ANOVA followed by Newman–Keuls post-test was also performed for other comparative analysis. Overall data was expressed as mean ± SEM (standard error mean) and a *p* value less than 0.05 was considered statistically significant. GraphPad Prism 5.0 (GraphPad Software, Inc., San Diego, CA, USA) was employed for statistical analyses.

## 3. Results

### 3.1. Assessment of Electroporation Competence and Its Effect on Survival Rate

**Figure 1:**
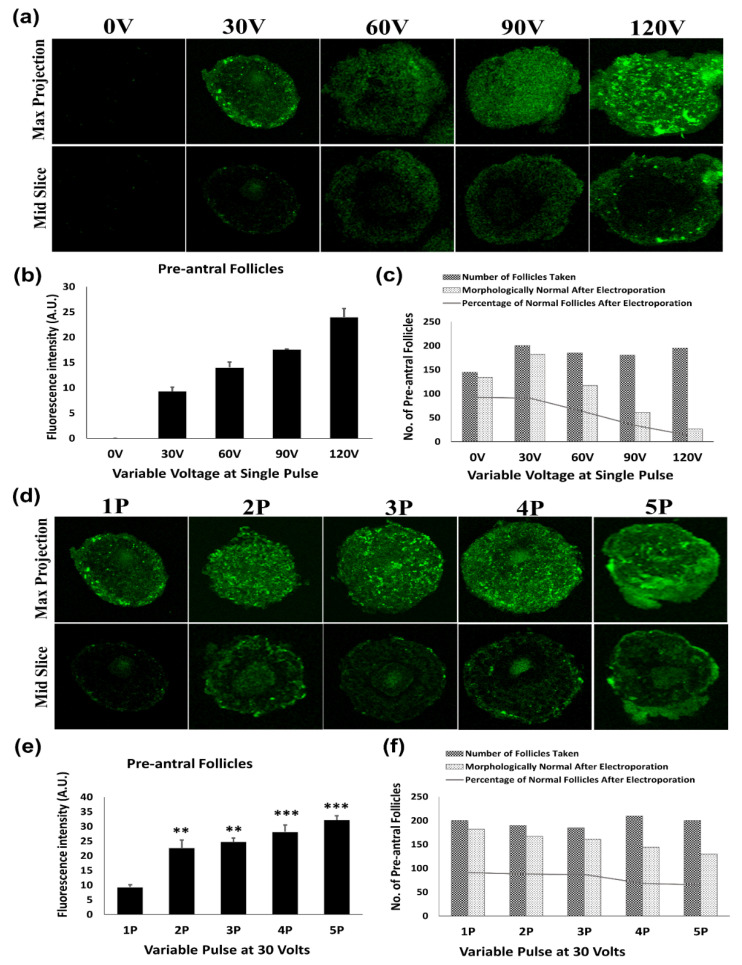
Photomicrograph depicts transfection efficiency of GFP through its fluorescence intensity in preantral follicles after electroporation. (**a**,**b**) Transfection of GFP increased gradually with increased voltage (30–120 V) within a single pulse. (**c**) However, this increased voltage caused deterioration in follicular morphology and reduced survival rate. (**d**,**e**) Consequently, multiple pulses of 30 volts at an interval of 10 s (1–5) were used; (**f**) where follicular morphology and survival was maintained until three consecutive pulses (i.e., 3 P of 30 V) were given with increased transfection efficiency. Beyond three pulses (4 and 5 P of 30 V), survival rate decreased (χ2 = 123.5, df = 8, *p* < 0.0001). Fluorescence intensity (arbitrary units) was quantified through ImageJ Software. ** *p* < 0.01 and *** *p* < 0.001 reflect a significant difference as compared to the single pulse of 30 V. Original magnification of photomicrographs was ×400.

**Figure 2:**
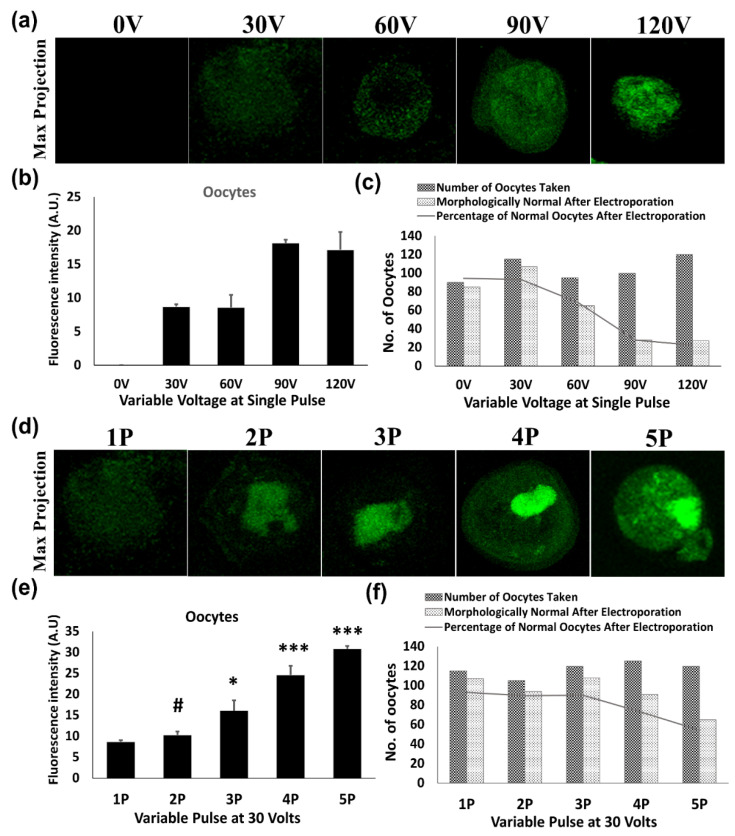
Pictorial representation showing optimization of GFP transfection through electroporation in mouse oocytes. (**a**–**c**) Increased voltage (60–120 V) at a single pulse reduced the survival rate of mice oocytes, (**d**–**f**) but lower voltage with multiple pulses showed improvement in the survival along with transfection efficiency up to three pulses (1–3 P of 30 V). After three pulses of 30 V (4–5 P), fluorescence intensity increased but with decreased survival (χ2 = 69.22, df = 8, *p* < 0.0001). The measurement of fluorescent intensity was done by ImageJ software. * *p* < 0.05 and *** *p* < 0.001 indicate significant differences compared to the single pulse of 30 V. # is non-significant. Original magnification of photomicrographs was ×400.

**Figure 3:**
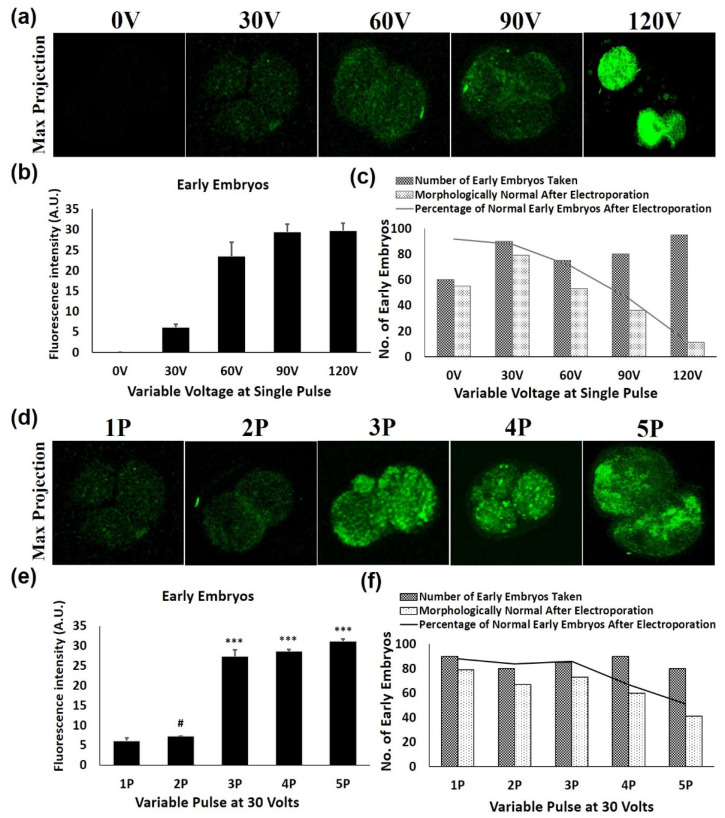
Representative image shows the transfection efficacy of GFP by electroporation in early mouse embryos. (**a**–**c**) A single pulse of more than 30 volts increased transfection efficiency but compromised the morphology of early embryos. (**d**–**f**) The multi-pulse 30 volt revealed improved transfection efficiency, but the survival decreased beyond four consecutive pulses (4–5 P). Thus, three pulses (3 P) at 30 V were observed to be optimal conditions for transfection of early embryos by electroporation (χ2 = 55.29, df = 8, *p* < 0.0001). *** *p* < 0.001 significant differences compared to the single pulse of 30 V. # is non-significant. Original magnification of photomicrographs was ×400.

**Figure 4:**
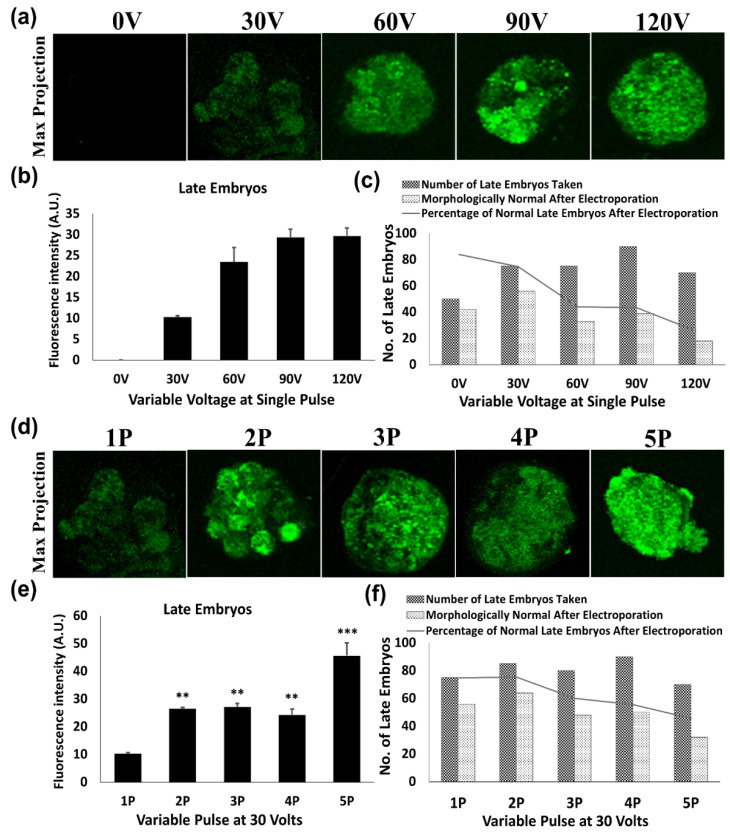
Assessment of GFP transfection by varying voltage and pulse in late mouse embryos following electroporation. (**a**–**c**) Higher voltages of more than 30 volts (60–120 V) at a single pulse resulted in the reduction in the survival of late mice embryos after electroporation; (**d**–**f**) on using the multi-pulse at 30 V, two pulses (30 V 2P) showed an optimal survival rate for the late embryos, which might be due to the zona hatching phenomenon (χ2 = 23.03, df = 8, *p* < 0.01). ** *p* < 0.01 and *** *p* < 0.001 indicate significant differences compared to the single pulse of 30 V. Original magnification of photomicrographs was ×400.

In order to measure electroporation transfection efficiency in follicles, oocytes and different embryonic stages, the strategy shown in Figure 1c was employed. Following transfection with pEGFP-C2 plasmid, the intensity of EGFP expression was measured under fluorescence microscopy in follicles, oocytes and different embryonic stages by merging all Z-stack slices after 48 h of electroporation. EGFP expression was feeble in all samples at single pulse of 30 V but with increasing voltage, a gradual increase in the EGFP expression was observed (Figure 1b, Figure 2b, Figure 3b and Figure 4b). However, this increased voltage steadily reduced the survival rate in all samples, as shown in Figure 1c. The survival percentage was calculated by (No. of follicles successfully grown after electroporation/No. of follicles taken × 100). Consequently, the voltage was fixed at 30 V and the number of pulses (2, 3, 4 and 5) were compared in order to obtain higher cell viability along with higher transfection efficiency. In follicles, increasing the pulse number from 1 to 3 led to a substantial improvement of transfection (*p* < 0.01) (Figure 1e); while a further increment of pulse number (Figure 1f) from 4 to 5 compromised the viability (68.57% for 4 pulses and 65.00% for 5 pulses) with a significant difference in the follicle viability (χ2 = 123.5; df = 8; *p* < 0.0001), which was quite evident through the altered follicle and oocyte morphology after electroporation. A strong EGFP signal was observed in the primary oocytes at 30 V, where higher voltages did not make much difference for EGFP expression of the primary oocyte within the pre-antral follicle. At the same time, multiple pulses of 30 V could increase EGFP expression of the oocyte within the pre-antral follicle (Figure 1a,d). Hence, electroporation parameters were standardized as per the cell viability.

Similarly to follicles, in oocytes and early embryos, electroporation efficiency was increased with increasing voltage. Upon increasing the voltage from 30 V to 120 V, a slight increase in transfection was achieved but a drastic reduction in the survivability of oocytes (93.04% to 22.50%) and early embryos (87.77% to 11.57%) was witnessed along with impairment in the embryonic development. However, EGFP showed a significant expression by applying 30 V of 3 pulses at an interval of 10 s in both oocytes (*p* < 0.05) (Figure 2e) and early embryos (*p* < 0.001) (Figure 3e). Furthermore, the Pearson chi-square for oocytes (χ2 = 69.22; df = 8; *p* < 0.0001) and early embryos (χ2 = 55.29; df = 8; *p* < 0.0001) indicated a significant change in the survival of these cells when compared between all the conditions but no significant change was observed in survival until three multiple pulses in oocytes (χ2 = 0.09814; df = 3; *p* = 0.9921) as well as in early embryos (χ2 = 0.1411; df = 3; *p* = 0.9865). Moreover, survival dramatically decreased from 90.00% to 54.16% (Figure 2f) and 85.88% to 51.25% (Figure 3f) for 3 to 5 pulses in oocytes and early embryos, respectively.

Therefore, these results suggest that 3 pulses of 30 V of 1 ms each at an interval of 10 s is the ideal condition for electroporation in follicles, oocytes, and early embryos, with no need to weaken or loosen the ZP layer.

We then wanted to further understand whether these optimized conditions would show similar transfection efficacy in late stage embryos as well. Morulae stage embryos (late embryos) (Figure 4) were also transfected with the *p*-EGFP plasmid. Although the viability of the embryos declined (74.66% to 25.71%) with higher voltages (30 V to 120 V), fluorescence intensities followed the same trend as observed during electroporation of early embryonic stages with corresponding voltages. However, the highest survival rate was achieved with two pulses at 30 V (75.29%) and almost 70% of the survived embryos subsequently developed into blastocysts. There was no significant difference in the fluorescent intensity among two and three pulse groups at 30 V, but the three pulse group exhibited 15.29% more mortality than the two pulse group (Figure 4f). The reason behind the higher mortality rate in the three pulse group may be due to the slight zonal hatching process during late embryonic stages that generally reduces and thins the ZP membrane prior to implantation.

### 3.2. Evaluation of In Vitro Oocyte Maturation after Electroporation

**Figure 5:**
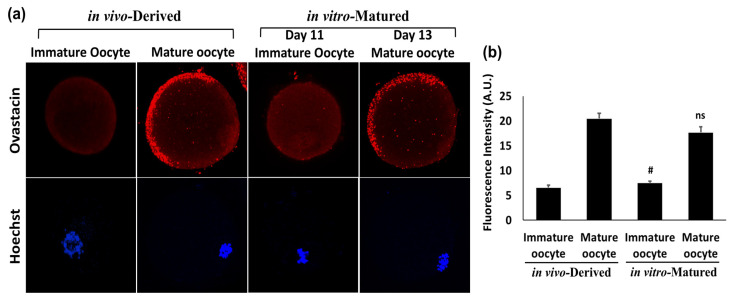
Authentication of an in vitro-matured oocyte obtained from a GFP electroporated preantral follicle through the immuno-localization of the oocyte maturation marker ovastacin. (**a**) Immature and mature oocytes derived in vivo were compared with in vitro-matured oocyte collected on day 11 and 13 from the preantral follicle after the electroporation with GFP, respectively. Ovastacin is known to localize throughout the cytoplasm of the immature mouse oocytes (germinal vesicle stage) and mostly concentrate at the cell periphery, whereas in MII mature oocytes, it localizes mainly in the microvillar domain of the oolemma. The expression pattern of ovastacin (red) was found to be similar for both the corresponding stages; in vivo-derived oocytes with in vitro-cultured oocytes from transfected preantral follicles. (**b**) Comparison of fluorescent intensities between in vivo derived vs. in vivo matured groups. # and ns indicate a non-significant difference in in vivo-derived vs. in vitro-matured groups, respectively. Original magnification of photomicrographs was ×400.

In order to check whether optimized conditions of electroporation (i.e., 3 pulses of 30 volts) does influence the oocyte maturation, immune-localization was done for the known oocyte maturation marker ovastacin. Preantral follicles after the electroporation with the pEGFP plasmid were grown in vitro and matured oocytes were obtained. Ovastacin, an oolemmal protein, has been shown to be expressed throughout the cytoplasm of the immature mouse oocytes (germinal vesicle stage) but it mostly concentrates at the cell periphery, whereas in MII mature oocytes, it gets localized mainly in the microvillar domain of the oolemma [20]. Therefore, expression of this maturation marker was assessed to compare in vivo-derived normal mature oocytes vs. in vitro-matured oocytes from preantral follicles after the electroporation. Ovastacin (red) localized almost equally on the oolemma and some parts of the cortical region in both in vivo-derived as well as in vitro-matured oocytes collected on day 13 of the culture (Figure 5a). In both cases of immature oocytes, i.e., in vivo-derived as well as in vitro-matured oocytes collected on day 11 of the culture from preantral follicles, ovastacin was less abundant and localized throughout the cytoplasm. As per the quantitative analysis of the fluorescent intensities, a slight decrease in the fluorescent intensity was observed for the in vitro-matured oocytes as compared to in vivo-derived oocytes, but this change was non-significant (Figure 5b). Thus, immuno-localization of ovastacin established that 30 volts of 3 pulses does not hamper the oocyte maturation process and hence, these are the ideal conditions for the in vitro transfection of follicles through electroporation.

### 3.3. Evaluation of Embryo Impairment Following Electroporation

**Figure 6:**
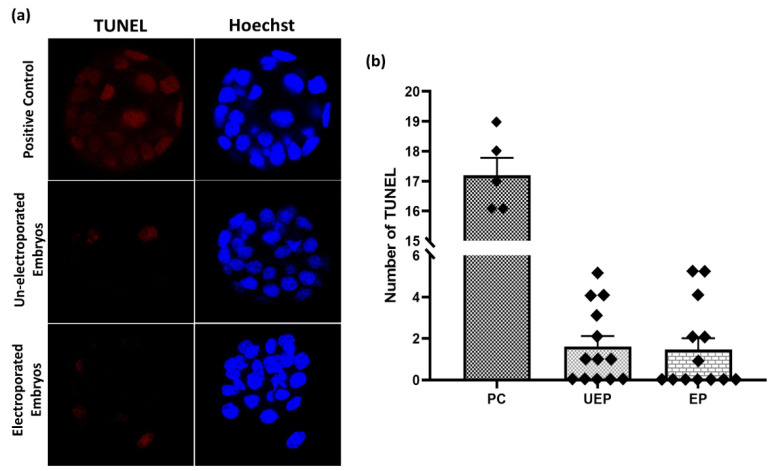
Assessment of in vitro embryonic development after the GFP electroporation through the TUNEL assay. Apoptotic nuclei in un-electroporated (UEP) vs. electroporated (EP) mouse embryos were observed by TUNEL assay, which labels the fragmented DNA. (**a**) Confocal microscopy showed no apparent difference in apoptotic nuclei (red) between electroporated and un-electroporated blastocysts. Positive control blastocysts were TUNEL-labeled after DNase treatment. Nuclei were stained with Hoechst 33342 (blue) in all groups. (**b**) Apoptotic nuclei number in each blastocyst. Original magnification of photomicrographs was ×400.

In order to assess the level of impairment in the electroporated embryos, the TUNEL assay was performed on normal blastocyst embryos collected in vivo as well as the in vitro-cultured blastocysts obtained from their subsequent morulae stage embryos after electroporation. The selected electroporation parameters for morulae embryos were 2 pulses of 1 ms at 30 V. As shown in Figure 6, positive control embryos showed abundant TUNEL-positive signal, while no significant difference was observed in the number of apoptotic nuclei between the in vitro-grown un-electroporated vs. electroporated embryo groups.

### 3.4. Electroporation of a miR-26a Inhibitor and Mimic in Preantral Follicles

**Figure 7:**
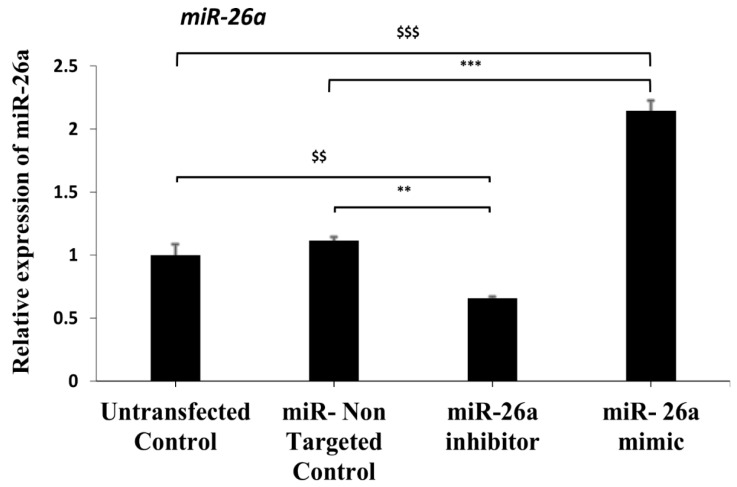
Utilization of the standardized technique for the electroporation and regulation of miR-26a. Expression of mmu-mir-26a in preantral follicles after transfection with miR-26a inhibitor and mimic. Preantral follicles were transfected with miR non-targeted control, miR-26a inhibitor and miR-26a mimic using 30 volts of 3 pulses at an interval of 10 s by electroporation. The expression of miR-26a was measured by qRT-PCR after 48 h of transfection and normalized by RNU6-6P expression. ** *p* < 0.01 and *** *p* < 0.001 indicate significant differences compared to the miR- non-target control group, whereas ^$$^ *p* < 0.01 and ^$$$^ *p* < 0.001 indicate significant differences compared to the untransfected control group.

To evaluate the efficiency of electroporation for the transfection of a miRNA inhibitor and mimic, a mmu-miR-26a inhibitor and mimic and a miR control were transfected in preantral follicles. As per real time PCR analysis, the miR-26a inhibitor-transfected follicles showed significant down regulation of mmu-mir-26a expression as compared to the miR- non-targeted control group (*p* < 0.01). Furthermore, the miR-26a mimic-transfected follicles showed highly significant upregulation of the expression of mmu-mir-26a as compared to the miR- non-targeted control group (*p* < 0.001). In addition, both the mmu-miR-26a inhibitor (*p* < 0.01) and mimic (*p* < 0.001) groups manifested efficient transfection through electroporation, when compared with the untransfected control group in which follicles were only subjected to electroporation conditions. Collectively, these results authenticated the effectiveness of the electroporation process in transfecting miR- inhibitors and mimics in the follicles (Figure 7).

## 4. Discussion

An in vitro follicle culture system has become a vital tool to study folliculogenesis, oogenesis and early embryonic development. It serves as a perfect model to explore the function of genes and thus creates an opportunity to understand the regulatory mechanism of the development of a follicle to a growing embryo. In the course of folliculogenesis, the oocyte attains maturation and is surrounded by the zona pellicuda (ZP). The ZP is a protective glycoprotein coating that encircles the plasma membrane of oocytes and early embryos before implantation [21]. This covering triggers the sperm to undergo acrosomal reaction and acts as a selective filter for them, only allowing sperm that have completed the acrosomal reaction. However, the ZP acts as a potential hindrance for transfection related studies in these cells [22]. Regardless, the usage of electroporation for the alteration of genetic material in the cells is being adopted by various laboratories as it is a relatively economical, safe and faster technique for the delivery of exogenous DNA plasmids and RNA. However, fine-tuning of parameters like voltage, pulse amplitude and pulse number needs to be standardized every time to achieve better transfection efficiency [23]. The transfer of foreign nucleic acid in oocytes and preimplantation embryos using electroporation was demonstrated successfully for the first time in 2002 [3]. However this initial study demonstrated the electroporation of rhodamine-conjugated dextran in ZP-removed oocytes and zygotes, but the removal of ZP usually hampers in vitro growth in these cells and also reduces their in vivo survival if transferred in pseudo-pregnant foster mothers due to their adhesive property [24,25]. A diffuse signal pattern of GFP was observed throughout the cytoplasm in the case of weak transfection with a single pulse, whereas increased transfection efficiency with multiple pulses (Figure 2b) promoted accumulation of GFP localization. Alternatively, acidic Tyrode’s solution was used to loosen the ZP for the transfection of these cells, which reasonably prevented the damage caused by electroporation. Subsequently, many studies achieved delivery of foreign genes to early embryos via electroporation by this zona loosening strategy [16,17]. However, application of caustic treatments is harmful and gradually compromises the developmental potential of embryos in vitro [17]. Contrarily, techniques involving transfection in intact zygotes require specialized devices that are not widely available [26,27]. To overcome these concerns, the present study established an easy transfection method for ZP intact oocytes and embryos, which can be done with any ordinary electroporation equipment.

A series of conditions were optimized for highly efficacious transfection in intact mice follicles, oocytes and various embryonic stages through electroporation while maintaining a high survival rate as well as uncompromised developmental potential. Firstly, a higher voltage for the electroporation of these cells was applied, but it triggered a fall in the survival rate and also changed the overall morphology. Hence the idea of implementing a higher voltage in these cells was dropped and an increased number of pulses at a lower voltage was determined. On applying 30 V of varying pulses, a gradual increase in the electroporation was achieved with high survival rates. However, more than three consecutive pulses of 30 V compromised the survival rate in the follicles, oocytes, and early embryos. Accordingly, 3 pulses of 30 V of 1 ms each at an interval of 10 s were observed to be the best electroporation conditions for highly efficacious transfection.

In the case of late embryos (morulae stage), a non-significant change in electroporation efficiency was observed by increasing the pulses from 2 to 3. Furthermore, two pulses of 30 V increased survival up to 75%, whereas beyond 2 pulses of 30 V decreased the percentage of embryo survival, as well as further development to the subsequent blastocyst stage. This might be due to the zona thinning and the blastocyst expansion process during late embryonic stages prior to implantation [28]. Moreover, the cell apoptosis assay was performed in order to validate the healthy embryonic development following electroporation. Apoptosis is a process by which defective cells are usually eliminated but it is not generally observed throughout normal embryonic development [29]. The initiation of apoptosis by chemical or physical injury during preimplantation embryonic development could lead to deletion or damage of important cell lineages and hence affect the normal embryonic development that may lead to embryonic malformation or abortion [17,30]. The results of the TUNEL apoptosis assay showed no significant difference in the apoptotic nuclei number of blastocysts developed in vitro from electroporated embryos in contrast with normal un-electroporated blastocysts grown in vitro. To authenticate the TUNEL apoptosis assay, a positive control experiment was also designed simultaneously, which showed ample TUNEL-positive nuclei. Thus, suggesting that these parameters are safe for intact embryo electroporation and do not induce any embryo impairment after electroporation.

In addition, proper maturation of the preantral follicles after electroporation with the selected conditions was successfully evaluated. Previous reports have already documented the change in the staining pattern of ovastacin during oocyte maturation [20]. As per the previously published results, in ovulated immature oocytes (germinal vesicle stage), ovastacin was observed throughout the ooplasm with enrichment at the oocyte periphery, whereas in the mature MII stage of oocytes, ovastacin concentrates in the microvillar domain of the oolemma antipodal to the nucleus. Hence, this marker was explored in our sequential experiments of in vitro oocyte maturation, and the observations were found to be similar during the in vitro transition of the germinal vesicle to mature MII oocyte, where ovastacin also re-oriented in the oolemma. Our results also showed successful manipulation of miRNA expression by transfection of the miR inhibitor and mimic in preantral follicles through electroporation. The expression of mmu-mir-26a showed a significant decrease in the miR-26a inhibitor-transfected follicles, while a considerable increase in the expression of mmu-mir-26a was observed in the miR-26a mimic-transfected follicles as compared to both vehicle control and non-targeted control.

## 5. Conclusions

In conclusion, this study offers an efficient transfection method using electroporation for intact mouse follicles that would help to provide a greater understanding during in vitro analysis of folliculogenesis. Concurrently, this transfection procedure is equally applicable for ZP enclosed oocytes and embryos and does not require the corrosive treatments for the weakening of ZP. Other advantages of this technique over other classical transfection methods are that it is simple, efficient, affordable, non-laborious and less toxic; at the same time, instantaneous co-delivery of multiple plasmids and molecular drugs is possible. Thus, these optimized conditions for electroporation can provide insights about the molecular mechanisms modulating folliculogenesis, oogenesis and embryogenesis.

Thus, these results validate the standardization of the electroporation technique for effective transfection and also opens a new vista for its potential application in assisted reproductive technologies (ART) in clinical settings.

## Data Availability

The data presented in this study are available within the article.
